# Bone Marrow Transplantation Results in Human Donor Blood Cells Acquiring and Displaying Mouse Recipient Class I MHC and CD45 Antigens on Their Surface

**DOI:** 10.1371/journal.pone.0008489

**Published:** 2009-12-31

**Authors:** Nobuko Yamanaka, Christine J. Wong, Marina Gertsenstein, Robert F. Casper, Andras Nagy, Ian M. Rogers

**Affiliations:** 1 Samuel Lunenfeld Research Institute, Mount Sinai Hospital, Toronto, Ontario, Canada; 2 Department of Obstetrics and Gynecology, University of Toronto, Toronto, Canada; 3 Department of Molecular Genetics, University of Toronto, Toronto, Canada; City of Hope National Medical Center, United States of America

## Abstract

**Background:**

Mouse models of human disease are invaluable for determining the differentiation ability and functional capacity of stem cells. The best example is bone marrow transplants for studies of hematopoietic stem cells. For organ studies, the interpretation of the data can be difficult as transdifferentiation, cell fusion or surface antigen transfer (trogocytosis) can be misinterpreted as differentiation. These events have not been investigated in hematopoietic stem cell transplant models.

**Methodology/Principal Findings:**

In this study we investigated fusion and trogocytosis involving blood cells during bone marrow transplantation using a xenograft model. We report that using a standard SCID repopulating assay almost 100% of the human donor cells appear as hybrid blood cells containing both mouse and human surface antigens.

**Conclusion/Significance:**

Hybrid cells are not the result of cell-cell fusion events but appear to be due to efficient surface antigen transfer, a process referred to as trogocytosis. Antigen transfer appears to be non-random and includes all donor cells regardless of sub-type. We also demonstrate that irradiation preconditioning enhances the frequency of hybrid cells and that trogocytosis is evident in non-blood cells in chimera mice.

## Introduction

Stem cell biology relies heavily on the specificity of surface markers as an initial determination of a cells identity after differentiation. Presentation of a predetermined set of markers characteristic of the desired cell type being investigated is required before more complex studies to determine cell function are carried out. *In vitro* studies can be used to determine cell function but the ability of a newly differentiated cell to successfully reverse the affects of a disease in an experimental animal model is the gold standard. Being able to identify donor cells in any animal model is critical for determining the extent and the mechanism of engraftment and differentiation.

Mouse models for bone marrow transplantation with hematopoietic stem cells (HSC) have become the paradigm for stem cells analysis since their discovery almost 50 years ago [1], [2]. With the discovery of embryonic and tissue specific stem cells, mouse models of disease are also often used to measure the differentiation and functional capacity of these cells. As with HSC transplants, embryonic or tissue specific stem cells are delivered to the diseased organ and successful engraftment and restoration of tissue function is measured post transplantation [3], [4], [5], [6]. Both intraspecies and xenograft models of disease have been successfully used to demonstrate the functional capacity of different types of human and mouse stem cells. Additionally, mouse models of human disease are ideal for determining the clinical significance of novel stem cell based therapies due to the incidence of similar transplantation issues that occur in a clinical setting such as graft rejection and, for bone marrow transplants, graft versus host disease (GvHD) [7], [8], [9], [10].

The analysis of HSC engraftment in most cases is straightforward because the donor cells can be easily recovered from the bone marrow as single cells and their contribution is measured by antibodies to donor specific cell surface proteins using flow cytometry. For solid organ transplants such as pancreas, spinal cord or heart, the delivery of cells and their subsequent identification and recovery after transplantation is more complex. Usually, immunohistochemical analysis of solid organs is required. The difficulty of obtaining high levels of engraftment, coupled with the difficulty of tissue recovery and analysis results in few positive events. Further complicating the interpretation of the data is that differentiation events in animal models have been attributed to differentiation, transdifferentiation, cell fusion or surface antigen transfer (trogocytosis), depending on the donor cells and the animal model being used.

We were interested in determining if cell fusion or surface antigen transfer plays a role in hematopoietic cell differentiation in NOD.CB17-Prkdc^SCID^ (NOD/SCID) mouse models. Using a standard SCID repopulating assay, we transplanted irradiated NOD/SCID mice with either the total nucleated cell (TNC) population or the donor blood cells were separated into Lineage positive (mature) cells or the Lineage minus (stem and progenitor) cells. The blood cells used were from human umbilical cord blood or human bone marrow. The use of human cells allowed us to distinguish between human cells (HLA:ABC+) and mouse cells (H2Kd+). Hybrid cells would be positive for both human and mouse antigens [11], [12].

Cell fusion involves the fusion of cell membranes, cytoplasm and the nuclei resulting in a hybrid cell that contains chromosomes and proteins from both cells [13]. Trogocytosis involves only the transfer of cell surface antigens without the transfer of DNA or cytoplasm and transdifferentiation is defined as the ability of a differentiated cell to change phenotype to an unrelated cell without undergoing a de-differentiation step [14], [15]. In order to differentiate between cell fusion, trogocytosis or transdifferentiation it is important to investigate the chromosome content and the surface antigen composition of the cell. Xenograft models are ideal for determining the extent of these three possible processes during blood cell engraftment and differentiation. Analysis of fusion or trogocytosis will result in a mix of human and mouse components in a single cell, while transdifferentiation will not.

Despite the long time use of bone marrow transplants in both the clinical and experimental setting, fusion or trogocytosis between blood cells has not been well documented. In contrast, cell fusion has been well studied in transplantation models used to explore the possible transdifferentiation of blood cells [16], [17], [18]. Moreover, trogocytosis has not been considered in the stem cell field mainly because its mechanism is unknown and its significance has been limited to antigen presentation involved in immune reactions [19], [20], [21], [22].

Our results reveal that the majority of engrafted human cells are indeed hybrid cells that are a result of surface antigen transfer (trogocytosis) and not cell-cell fusion. Furthermore our results demonstrate that trogocytosis is much more widespread than previously thought resulting in the transfer of host (mouse) MHC Class I and CD45 antigens to donor (human) cells. We discuss the implications of these results on the interpretation of blood cell based therapies to treat blood and non-blood disorders.

## Results

### Xenograft Bone Marrow Transplants Results in H2Kd+HLA+ Hybrid Cells

**Figure 1:**
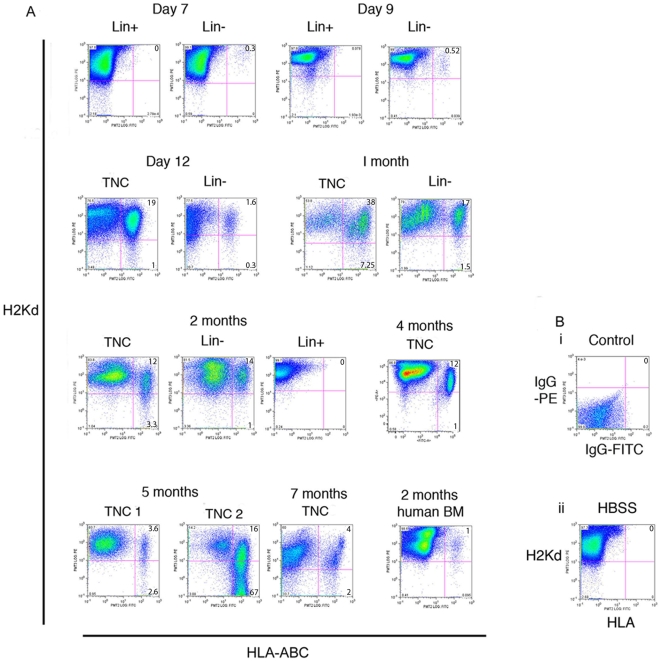
Engrafted human cells express mouse H2Kd class I antigens. (A) Flow cytometry results from mice engrafted with human blood cells analysed at different times post-transplantation. Cells were analysed by FACS with anti-H2Kd-PE antibody to identify mouse MHC-class I proteins and anti HLA-ABC-FITC antibody to identify human HLA class I proteins. The majority of human cells found in positively engrafted animals were double positive for H2Kd and HLA-ABC. The Lin+ population are non-engrafting mature blood cells while the Lin- population are enriched for hematopoietic stem cells. TNC are the unfractionated leukocyte population. (B) i) IgG negative control. ii) Mouse injected with HBSS/no cells followed by bone marrow analysis after 8-weeks using the same antibodies used to detect human cell HLA and mouse cell H2Kd. The HLA antibody does not cross react with mouse cells.

Irradiation of mice followed by intravenous infusion of human hematopoietic stem cells is a common model for the study of bone marrow transplantation. The use of immune compromised mice such as the NOD/SCID mouse allows for successful xenografts and has been instrumental in the development of bone marrow transplant regimes for use in patients. With reports that blood cells are capable of fusing with cells of the liver, Purkinje cells and lung cells [23], we sought to determine if blood cells are also capable of fusing amongst themselves. We also wanted to determine the extent of trogocytosis between donor and recipient cells in a xenograft model as this process can be interpreted as a cell fusion event. The xenograft model is ideal for this study as human and mouse blood antigens can easily be distinguished using flow cytometry with species specific antibodies to MHC class I or HLA antigens followed by FISH with mouse and human specific pan-centromeric probes. Flow cytometry allowed us to detect double positive cells even if their occurrence was rare. In this study we report on the results from 119 mice. TNCs, Lin- or Lin+ cells from human umbilical cord blood or human bone marrow were used for our xenograft studies. NOD/SCID mice were prepared and transplanted as per standard protocols [11], [24], [25]. Our standard engraftment time of 8–10 weeks was used in the preliminary set of results. Mice were transfused with 3.5–8 million TNCs or 150,000 Lin- cells or 150,000 Lin+ cells. Mice were assessed at 1 week post transplantation to 7 months post transplantation. All mice were assessed for human cells using an antibody against the human leukocyte antigens-A,B,C (HLA) and mouse cells using an antibody to the mouse MHC H2Kd (H2kd) antigen that is specific for NOD/SCID mice. 88% of the umbilical cord blood (UCB) -transplanted mice (76/86) were positive for HLA+ cells and all of these positive mice (76/76) contained hybrid cells (H2Kd+HLA+) (Figure 1A). IgG negative controls (mouse: TNC, 2 months) and sham treated mice (mouse: HBSS, 2 months) were always negative for HLA antibody reactions (n = 8) (Figure 1B). Mice that received Lin+ cells (n = 12) were also negative for HLA-ABC staining. For all positively engrafted animals except one, >80% of the human cells (HLA+) isolated from the recipient mouse bone marrow co-expressed mouse H2Kd antigen (HLA+H2Kd+). One mouse transplanted with human UCB cells and assayed at 5 months had a high level of human blood cell engraftment (82%) and was the only mouse where the majority of human cells did not also contain the mouse H2Kd antigen. It is possible that the high percentage of human cells meant that the mouse cells available for fusion or trogocytosis were limited. Human bone marrow was also tested using the same transplant conditions (n = 13), with mice assessed at 1 and 2 months. Engraftment occurred in 11/13 mice and hybrid cells were also observed in all engrafted mice (representative mouse: Figure 1A, last panel, 2 month data).

The H2Kd and HLA-ABC antibodies used in this study were tested for cross species reactions and non-specific reactions using either mouse BM or human UBC blood or a 50:50 mixture of mouse and human blood cells (Figure S1). Results indicate that the anti-HLA antibody only bound human cells while the anti-H2Kd antibody only bound mouse cells as expected. All other mouse or human specific antibodies used in this study were also tested for non-specific binding and demonstrated species specificity (data not shown).

### FISH Analysis Reveals That Trogocytosis and Not Fusion Occurs between Human and Mouse Blood Cells in the Xenograft Model and That the Transfer of HLA/MHC Class I Antigens Is Unidirectional

**Figure 2:**
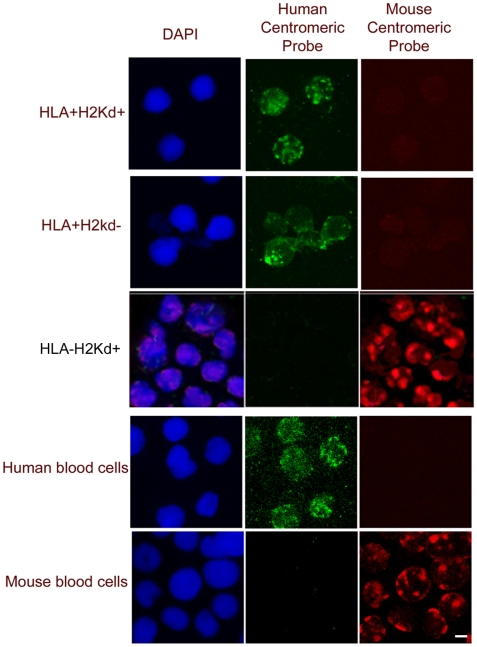
Confirmation of trogocytosis. Pan-centromeric probes were used to detect the presence of mouse and human chromosomes in the H2Kd+/HLA+ cells to ascertain the occurrence of fusion. FACS-sorted cells from an engrafted mouse were analysed by FISH while DAPI identified all nuclei. HLA+H2Kd+ and HLA+H2Kd- cells tested positive for human chromosomes and negative for mouse chromosomes. FISH results from FACS-sorted HLA-H2Kd+ cells from the same mouse demonstrated these cells contain only mouse chromosomes and not human chromosomes. These results confirm that trogocytosis rather than fusion between cells was the cause of the hybrid cells and this also confirms that HLA/MHC Class I protein is transferred in the recipient to donor direction only. Scale bar = 5 µm

The observation of such high levels of hybrid cells post engraftment was unexpected. Our hypothesis was that fusion or antigen transfer between blood cells may occur but we did not expect to observe nearly 100% involvement of the donor cells. Therefore, we sought to confirm if the mechanism is cell fusion and/or trogocytosis through fluorescent in situ hybridization (FISH). Pan-centromeric species-specific probes were used to ensure the random loss of some chromosomes that can occur in cell hybrids would not produce a false negative result. Post transplant bone marrow cells that were HLA+H2Kd+ (hybrid) HLA+H2Kd- (human only) or HLA-H2Kd+ (mouse only) were collected by FACS and subjected to FISH (Figure 2). The hybrid cells (H2Kd+HLA+) and the cells with HLA but not H2Kd, contained only human chromosomes and the non-hybrid mouse cells (H2Kd+HLA-) from the same mice were positive for mouse chromosomes only. Mouse bone marrow and human UCB cells were used to confirm that the mouse and human probes were species specific and that no cross reaction or non-specific reactions occurred. Karyotyping of the H2Kd+HLA+ cells confirmed our FISH results that only human chromosomes were present (data not shown).

The positive results we obtained showing the presence of both human and mouse surface antigens without the presence of mouse chromosomes strongly indicate that cell surface antigens are capable of transferring from one cell to another. Our FISH results confirm that trogocytosis is unidirectional as the H2Kd+HLA+ hybrid cells only contained human chromosomes. This result shows that the human cells acquired mouse surface proteins through trogocytosis. If trogocytosis also occurred in the other direction (human antigens to mouse cells) then we would have observed hybrid cells containing mouse chromosomes. Taken together, this data demonstrates that the donor human cells acquire the recipient mouse MHC antigens through trogocytosis and not fusion.

### CD45 Is Involved in Trogocytosis and Is Capable of Bi-Directional Transfer

**Figure 3:**
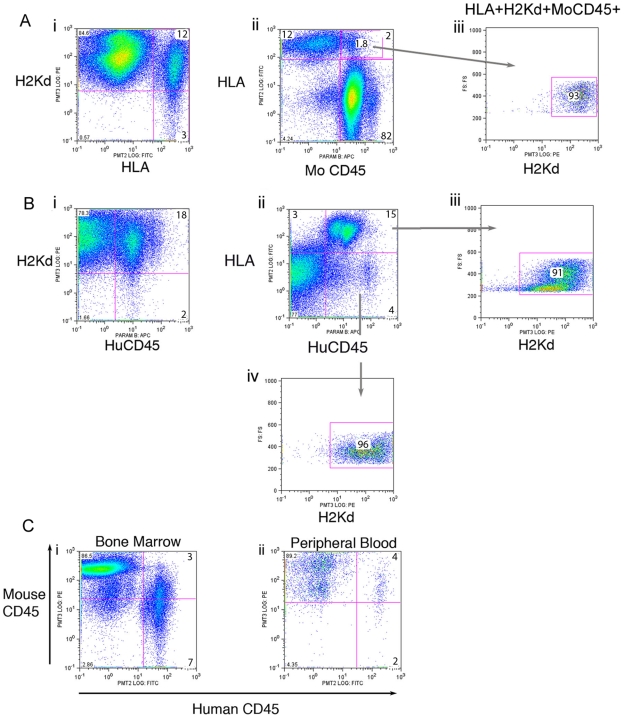
CD45 is involved in trogocytosis. Mice engrafted with human cells were analysed for HLA, H2Kd, mouse CD45 and human CD45 using flow cytometry. (Ai) A representative mouse demonstrated ∼15% HLA+ cells within the bone marrow with the majority also being H2Kd+. (Aii) 2% of the HLA positive cells were also mouse CD45+ indicating that mouse CD45 does transfer to human cells although at a lower frequency than H2Kd. (Aiii) The HLA+mouseCD45+ cells were also positive for H2Kd+ (H2Kd+HLA+mouseCD45+). The FISH data confirmed that H2Kd transfers to human cells but HLA does not transfer to mouse cells, therefore, these triple positive cells arise from the co-transfer of mouse CD45 and H2Kd to human cells. (Bi) From the same mouse there were more human CD45+ cells (20%) then HLA+ cells (15%) indicating that some of the human CD45+ cells are HLA negative. (Bii) Double staining for human CD45 and HLA revealed that 4% of the CD45+ cells were HLA negative. (Biii) 91% of the HLA+humanCD45+ cells were also positive for H2Kd. (Biv) Similarly, 96% of the HLA-humanCD45+ cells were also positive for H2Kd. Because of the high level of transfer of H2Kd to the human cells in the graft the data indicates that both human and mouse CD45 are involved in bi-directional trogocytosis. (C) Co-staining (i) Bone marrow cells and (ii) Peripheral blood cells from an engrafted mouse with mouseCD45 and humanCD45 antibodies revealed that the double stained cells contained reduced levels of mouse CD45 versus the mouse cells that were negative for human CD45. It is possible that hybrid cells can utilize both species of CD45 and regulate the levels in order to maintain cell homeostasis.

Since the bone marrow contains mostly CD45+ blood cells, it is conceivable that the CD45 antigen may be involved in trogocytosis. Although the bone marrow is comprised mainly of CD45+ blood cells we cannot assume that all of the HLA+H2Kd+ hybrid cells are also CD45+. A subset of the mice engrafted with human blood cells that were analysed for HLA+H2Kd+ cells were also examined for mouse CD45 and human CD45 to determine if these surface antigens are involved in trogocytosis. In the previous experiments we determined that H2kd is transferred to the donor human cells but there is no reciprocal transfer with HLA to mouse cells. For the experiments done here, the bone marrow cells from engrafted mice were stained with H2Kd-PE, HLA-FITC and CD45-APC (either mouse or human) (n = 16 with UCB and n = 8 with human bone marrow: 1–4 months post transplantation). All mice studied showed similar results. The representative data in Figure 3 was from a mouse that received UCB TNC and was analysed 2 months post transplantation. This mouse has 15% HLA+ cells of which 12% are H2Kd+HLA+ (Figure 3Ai). We observed that only 2% of the HLA positive cells were also mouse CD45+ (Figure 3Aii) and these cells are also positive for H2Kd (H2Kd+HLA+mouseCD45+) (Figure 3Aiii). Since H2Kd is transferred to human cells at a high frequency, these H2Kd+HLA+mouse CD45+ cells are most likely due to both mouse CD45 and H2Kd being transferred to the human cells and that mouse CD45 transfer occurs at a much lower frequency than for H2Kd.

When the bone marrow cells from the same mouse was stained for human CD45 we observed more human CD45+ cells than HLA+ cells (20% versus 15%) (Figure 3Bi) indicating that some of the human CD45+ cells must be HLA negative. This was confirmed when the same bone marrow cells interrogated for human CD45 and HLA. Approximately 4% of the human CD45+ cells were HLA negative (Figure 3Bii). HLA+humanCD45+ cells were investigated for H2Kd revealing that 91% were triple positive (Figure 3Biii) confirming that H2Kd is transferred to the human blood cells. When the 4% HLA- humanCD45+ cells were examined for the presence of H2Kd antibody, we could demonstrate that 98% of these cells have H2Kd on their surface (Figure 3Biv). There are two possible explanations for our observation. It is possible that H2Kd transferred to the huCD45 cells and at the same time these cells lost their HLA antigens but this is unlikely and the more parsimonious explanation is that human CD45 is capable of transferring to mouse bone marrow cells. Taken together the data reveals that CD45 is capable of bi-directional transfer although at a low frequency.

Bone marrow cells from mice engrafted with human cells were simultaneously stained with anti-mouseCD45 and anti-humanCD45. Cells that were mouseCD45+humanCD45- versus mouseCD45+human CD45+ had higher PE- fluorescent intensity (mouseCD45: Y-axis) indicating that they contained more mouse CD45 antigen/cell (Figure 3Ci). Because fluorescence intensity is directly proportional to the amount of antigen on the cell surface, this suggests that the hybrid cells (mouseCD45+humanCD45+) will regulate their total CD45 antigen levels by down regulating mouse CD45 due to the presence of human CD45 on the surface. A similar result was observed for peripheral blood cells (Figure 3Cii).

### Trogocytosis Is Not Due to Donor Macrophages

**Figure 4:**
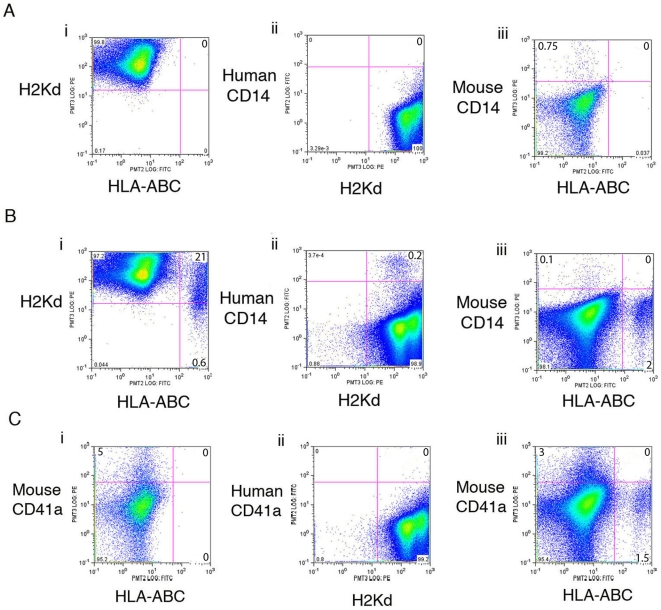
Mouse CD14 and CD41a do not transfer to human donor cells. (Ai) A non-engrafted mouse confirms that anti-human HLA-ABC and (Aii) anti human CD14 antibodies do not cross react with mouse cells. (Aiii) Anti-mouse CD14 identifies mouse macrophages in the bone marrow. (Bi) Bone marrow from an engrafted mouse contains a high percentage of human cells (HLA+) that are also H2Kd+. (Bii) Human CD14+ cells are also H2Kd+, confirming the transfer of H2Kd to human macrophages and (Biii) the absence of HLA+ cells co-expressing mouse CD14 indicates that unlike H2Kd and CD45, CD14 is not involved in trogocytosis. (Ci) In order to test the involvement of platelets in trogocytosis we tested for the transfer of CD41a. Bone marrow from a non-engrafted mouse stained with anti-HLA antibody and anti mouseCD41a antibody was negative for HLA but demonstrated that 5% of all bone marrow cells are mouse CD41a positive. (Cii) Bone marrow from the same engrafted mouse as used for CD14 detection reveals that the bone marrow was negative for human CD41a, indicating human platelets are not produced in NOD/SCID mouse. (Ciii) The absence of HLA+ cells co-expressing mouse CD41a confirms that CD41a is also not involved in trogocytosis.

Trogocytosis has been reported to involve antigen presenting cells such as macrophages. Therefore, we investigated the extent that macrophages, which express CD14, may contribute to our observations. No HLA+ or human CD14+ cells were detected in non-engrafted mice (negative control) (Figures 4Ai, Aii). Mouse CD14+ cells were observed as expected (Figure 4Aiii). In positively engrafted mice double positive H2Kd+HLA+ bone marrow cells were observed (Figure 4Bi). When the bone marrow from these mice were co-stained for H2Kd and human CD14, all of the human CD14+ cells were also positive for H2Kd antigen as expected as H2Kd transfers readily to the human cells (Figure 4Bii). Since the humanCD14+H2Kd+ cells represent a subset comprising only 7.2% of the of the hybrid cells (H2kd+HLA+), this indicates that phagocytosis by human macrophages are not solely responsible for the high rate of H2Kd+/HLA+ hybrid cells observed in the grafts. However, we cannot discount that macrophage phagocytosis is not the mechanism responsible for the appearance of this small subset of huCD14+/H2Kd+ hybrid cells. Figure 4Biii reveals that none of the HLA+ cells were positive for mouse CD14 indicating that mouse CD14 is not transferred to human cells.

### Platelets Are Not Involved in Trogocytosis Events

Previous studies have indicated that trogocytosis is specific to T-cells and antigen presenting cells, yet the data presented in this study indicates that many different blood cells are involved. We investigated if platelets are involved in the trogocytosis in our model by investigating if the CD41a antigen is transferred from the recipient cells to donor cells. Platelets are a fairly abundant cell type found in the bone marrow and should provide sufficient antigen (CD41a) to be involved in trogocytosis. Using the same mice used for CD14 detection, mouse CD41a+ cells were detected in the non-engrafted mouse (negative control) but no HLA+ cells were present, as expected (Figure 4Ci). However, engrafted mice did not contain any human CD41a+ cells (Figure 4Cii), indicating the human UCB cells were not capable of differentiating into platelets in our SRC assay. Therefore, we could not conclude if H2Kd is transferred to human platelets. Engrafted mice stained with antibodies to HLA and mouse CD41a indicate that as observed for mouse CD14, mouse CD41a does not transfer to human cells as no double positive cells were observed (Figure 4Ciii). This result suggests that not all recipient blood cells are involved and that trogocytosis is regulated in the xenograft model rather than occurring due to the close association of randomly paired cells.

### Location and Timing of Trogocytosis Indicates a Role for the Bone Marrow and Reveals the Involvement of Hematopoietic Stem and Progenitor Cells

**Figure 5:**
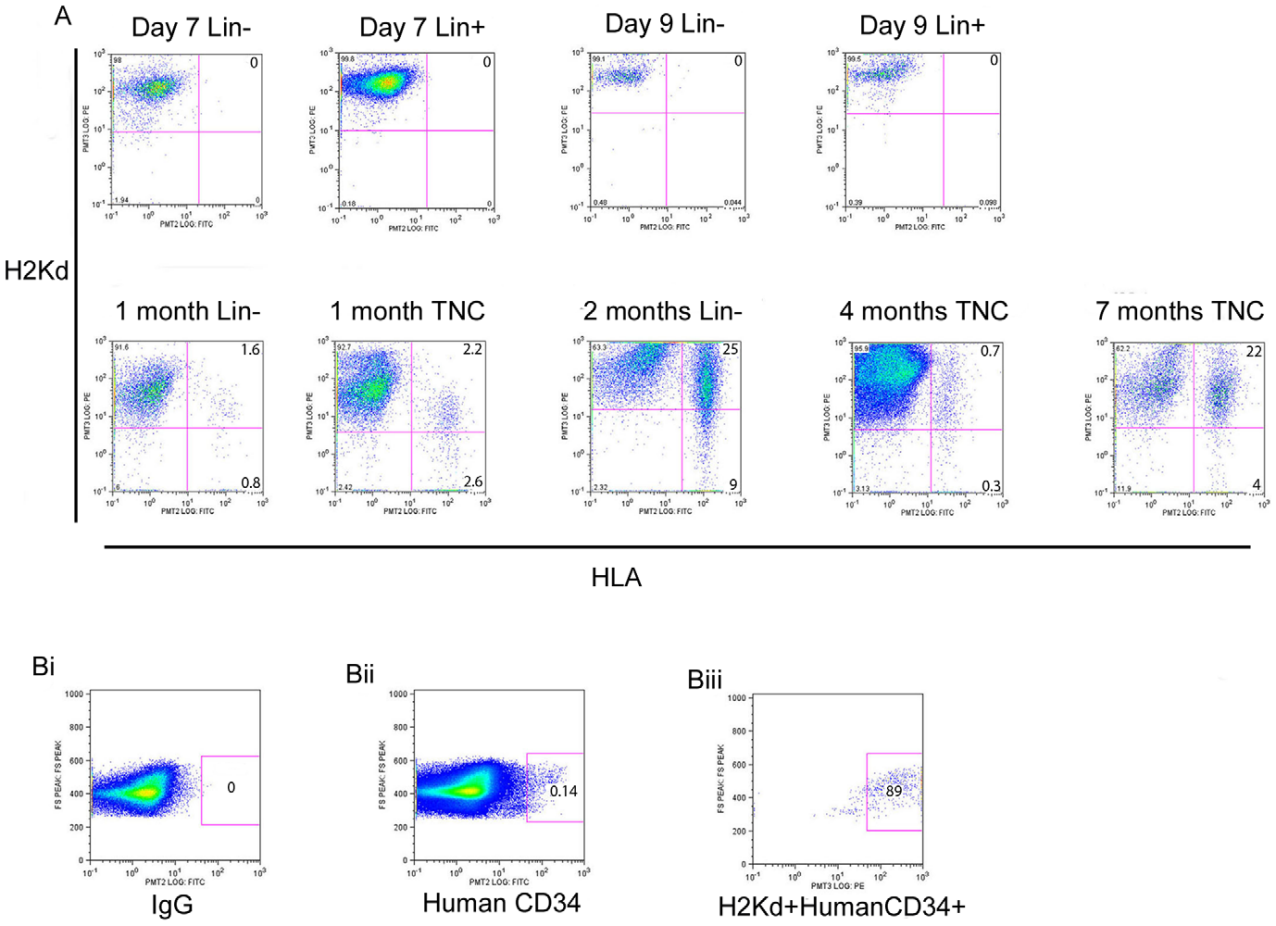
Trogocytosis occurs in the bone marrow and involves CD34+ cells. Peripheral blood from animals engrafted with Lineage negative (Lin-) or total nucleated cells (TNC) were double positive for human HLA and mouse H2Kd after 1 month post transplantation. Since the bone marrow but not the peripheral blood of day 7 and day 9 mice were positive for hybrid cells (compare to Figure 1), this suggests the human donor cells must first migrate to the bone marrow and engraft before trogocytosis can occur. Mice that received Lineage positive (Lin+) did not display hybrid cells. (Bi) CD34+ human cells make up a small population of engrafted cells (0.14%) but over 89% of them are also H2Kd+ indicating trogocytosis involves hematopoietic stem and progenitor cells.

In this report all donor cells were injected into the tail vein of the mice, which requires migration of these cells to the bone marrow in order to achieve long-term engraftment. It is possible that the donor cells encounter recipient cells in the peripheral blood (PB) where trogocytosis could occur between mature blood cells. In order to determine if the PB is the location of trogocytosis, we analysed peripheral blood from a subset of the animals presented in Figure 1. We tested animals transplanted with Lin- cells or Lin+ cells and could not detect human cells (HLA+) in the peripheral blood at 7 or 9 days post transplantation, but were able to observe human cells in the PB from 1 month to 7 months post transplantation mice (Figure 5A). The PB results for the 1–7 month post transplantation group were identical to that observed with their bone marrow cell analysis (Compare to Figure 1). The absence of human cells or hybrid cells in the PB of animals given Lin- or Lin+ cells at days 7 and day 9 post transplantation suggests that trogocytosis does not occur in the peripheral blood. Coupled with the observation that when the Lin+ are transplanted no hybrid cells are ever observed, suggests the trogocytosis occurs in the bone marrow.

Because trogocytosis occurs after the migration of donor cells to the bone marrow, this suggests stem and progenitor cell involvement. We tested for this using FACS, CFU and LTC-IC assays. First, we tested for the transfer of H2Kd to human CD34+ cells in engrafted NOD/SCID mice. Human CD34+ cells in the SCID repopulating assay represent a rare population. 4-months post transplantation bone marrow was flushed from a mouse transplanted with 1×10^8^ human TNC cells. The mouse was positive for human CD34+^hi^ cells (0.14%) and 89% of these cells were also H2Kd+ indicating the transfer of mouse H2Kd to human CD34+ cells (Figure 5B).

The CFU assay allowed use to confirm that hematopoietic progenitor cells are part of the hybrid cell (H2Kd+HLA+) population and also allowed us to confirm that trogocytosis and not cell fusion is the means for hybrid cell production. It is possible that a cell-cell fusion event could result in the translocation of the mouse H2Kd gene to a human chromosome, which results in the expression of the H2Kd protein on the hybrid cells. These translocation events would not be detected by our FISH analysis using centromeric probes. To confirm that the mouse:human hybrid cells did not contain any mouse DNA flow cytometry sorted and collected H2Kd+HLA+ cells were grown in CFU cultures that allowed the cells to undergo proliferation. Cell proliferation will result in the loss of H2Kd antigen on the cell surface if there is no mouse gene present to be transcribed. H2Kd+HLA+ cells were grown in CFU medium containing either mouse recombinant growth factors or human recombinant growth factors for 18 days. Control unsorted BM cells from mice that did not receive any human cells grew in medium with recombinant mouse growth factors but not in CFUs with human factors, while human UCB cells grew in both media (n = 2, each condition). Mouse-derived H2Kd+HLA+ cells were collected from mice at 2 weeks, 4 months or 5 months post-engraftment (n = 2, each condition). The hybrid cell displayed the same CFU growth properties as the human control cells. They grew well in media containing human growth factors or mouse growth factors, supporting the hypothesis that the cells contain only human DNA (Table 1). Furthermore, the cells from the hybrid-cell CFUs were collected post culture and stained with anti-HLA antibody and anti-H2Kd antibody. The cells were only positive for the human antigen (HLA+H2Kd-) having lost the H2Kd protein during cell culture (data not shown). Therefore culture resulted in the loss of the H2Kd protein as would be expected if the cells did not contain any mouse DNA.

**Table 1:** Hybrid cell contain blood progenitor cells as demonstrated by positive CFU growth.

CONTROLS	H4344*	M3434*
Mouse BM	0	23
Human UCB	44	28
HLA+/H2Kd+		
2weeks	28	15
4 months	40	65.5
5 months	29.5	35

* Colonies per 5000 cells/plate n = 2

Human blood cells are capable of forming colonies in medium containing human or mouse growth factors, while mouse blood cells are only capable of surviving in medium containing mouse growth factors. Hybrid cells (HLA+/H2Kd+) demonstrate growth characteristics of human blood progenitor cells. Cells from these CFUs were collected after 18 days in culture and stained for flow cytometry analysis with anti-H2Kd antibody. All cells that were originally H2Kd+/HLA+ at the beginning of the CFU culture lost the H2Kd antigen after the culture period indicating that the cells did not contain the H2Kd gene.

In order to confirm the involvement of donor stem and early progenitor cells, H2Kd+/HLA+ blood cells were collected from the bone marrow of engrafted mice (2 months post transplantation with UCB) and tested in a Long-Term Culture-Initiating Cell (LTC-IC) assay for the detection of stem/early progenitor cells (n = 2, each cell sample). Control cells included Lin- UCB cells that were plated at 5000 cells/LTC-IC and yielded 44 and 37 colonies/plate. H2Kd+/HLA+ cells produced 81 and 87 colonies per 1×10^5^ cells /LTC-IC assay. The presence of LTC-IC positive colonies from the H2Kd+/HLA+ bone marrow cells indicates trogocytosis includes the stem/progenitor cells.

Although CD34+ donor cells acquired H2Kd antigen on their surface the CFU-FACS experiment suggests that it is unlikely that CD34+ cells were able to retain the mouse antigens on their surface throughout cell proliferation and maturation. Taken together our data demonstrates that trogocytosis is an ongoing event that occurs in the bone marrow and the long-term engraftment of human blood stem cells in the mouse bone marrow provides for a constant supply of progenitor and maturing human blood cells that are involved in trogocytosis, which is why we can observe hybrid cells 7 months post engraftment.

### Studies of Chimera Animals Reveal That Blood Cell Trogocytosis Is Enhanced by the Irradiation Pre-treatment of Recipient Mice During Bone Marrow Transplantation

**Figure 6:**
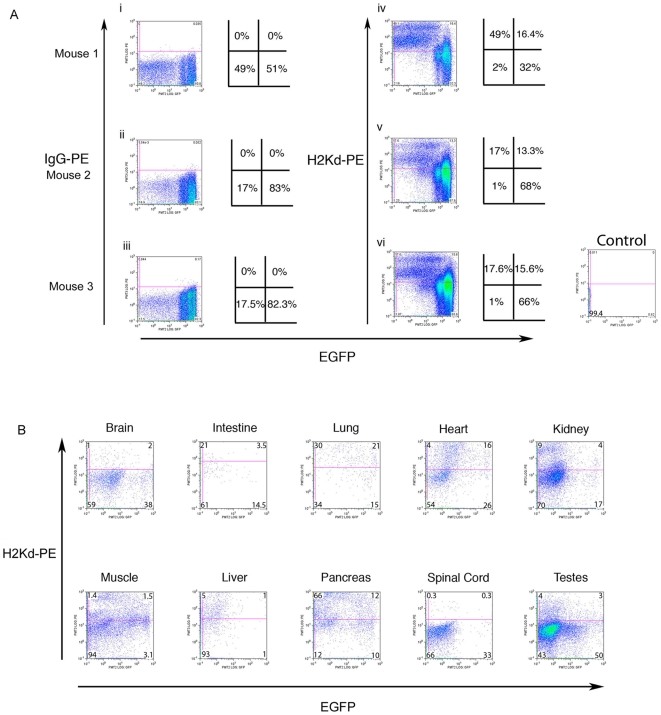
H2Kd is transferred between blood cells and non-blood organs of chimeric mice. Chimeric mice were generated from C57BL/6 EGFP ES cell line #12 with BALB/cAnNCrl morula. (A) The mouse blood cells displayed a range of EGFP expression levels. The voltage settings for the detectors were adjusted to clearly display the cells expressing low levels of EGFP as well as the high expressing cells, with negative cells gated close to the Y-axis. Adjacent pictomicrographs show the percentage of cells in each quadrant. The first column (i,ii,iii) represents the IgG-PE control and displays the EGFP+ cells. The second column (iv,v,vi) represents the same cells co-stained with H2Kd-PE. Double positive cells (H2Kd-PE+, EGFP+) are cells that have undergone trogocytosis of the H2Kd antigen. Observations of EGFP expressing cells that also express H2Kd was demonstrated by the shift of the population upward into the top right hand quadrant. Trogocytosis positive cells are 16.4%, 13.3% and 15.6% of the total bone marrow cells in the three mice respectively. Control; mouse blood cells from Balb/c mouse (EGFP-). B) Tissues from chimeric mouse #2 were analysed by flow cytometry. Tissue samples were stained with H2Kd-PE and flow cytometry was used to identify H2Kd-PE+ and EGFP+ cells. The percent chimerism for each organ was determined by EGFP+ cells and the amount of cells involved in trogocytosis was determined as the percentage of double positive cells (EGFP+, H2Kd-PE+) of the total EGFP+ cells. Tissues with high blood flow (heart and lung) have the highest levels of trogocytosis. The results are summarized in Table 2.

Previous studies have demonstrated that trogocytosis occurs during the formation of the immune synapse during routine immune cell interactions [26]. We have demonstrated that during bone marrow transplantation using an irradiation pre-conditioning the frequency of trogocytosis and the different types of cells involved greatly increases. In order to determine if trogocytosis between blood cells is a result of the irradiation pre-conditioning, we generated chimeric animals from two strains of mice with different MHC haplotypes. C57BL/6 ES cells (H2Kb) marked with enhanced green fluorescent protein (EGFP) transgene (C57BL/6-EGFP) and BALB/c mice (H2Kd). This study allowed for the production of animals with mixed HLA type blood cells without using irradiation. Thus, positive trogocytosis was determined by the presence of EGFP+/H2Kd + cells. Three chimeric mice were chosen based on their range of coat colour chimerism which was 30%, 50% and 80% respectively. Blood cell analysis for all three mice is depicted in Figure 6A. Blood cells and cells from tissues in the chimeric mice displayed a wide range of EGFP signal intensities. In order to detect the low and high expressing cells the voltage for the FACS detector was set to detect and display both low EGFP and high EGFP signals at the expense of keeping the negative cells on the Y-axis (see Materials and Methods for voltage settings). Original FACS plots with percentages of cells are shown along with pictomicrograph representation for positive (weak to strong expressing cells represented in the right side quadrants). IgG-PE isotype control is represented in Figure 6Ai,ii,iii. Note that EGFP cells can be observed in the IgG-PE isotype control and gives us a clear indication of the chimera contribution from the C57BL/6 blood cells. The blood cell chimerism does not reflect the coat colour chimerism with mice having 51%, 83% and 82.3% C57BL/6-EGFP blood cell contribution, respectively. We did observe hybrid EGFP+H2Kd+ cells, ranging from 13.3% to 16.4% of the total blood cells (Figures 6Aiv,v, vi). This experiment supports data from the literature stating trogocytosis occurs naturally, with this experiment specifically demonstrating involvement of the class I MHC(H2Kd) antigens. The frequency of trogocytosis in the blood of these chimeras is lower than in the xenograft studies but within the range expected for the previously described role for trogocytosis involvement in immune surveillance. This data indicates that preconditioning radiation used in the bone marrow transplant studies may increase the number of cells involved in trogocytosis.

**Table 2:** The percentage of cells involved in trogocytosis varies with the organ and is not related to the extent of tissue chimerism.

Tissue	% Tissue Chimera^a^	% Trogocytosis^b^
	(EGFP+ )/(total cells)	(EGFP+, H2Kd+ )/(all EGFP+ cells)
Brain	40	5
Intestine	18	19
Lung	36	58
Heart	41	39
Kidney	21	19
Muscle	5	30
Liver	2	50
Pancreas	22	54
Spinal Cord	33	1
Testes	53	5

Trogocytosis positive cells are identified as cells expressing the EGFP gene and the H2Kd gene. The extent of trogocytosis varies widely amongst the different organs and is more frequent in organs that have high blood flow. This experiment shows trogocytosis in only the one direction (Balb/c to C57Bl/6).

a) Chimerism is based on the percentage of EGFP+ cells.

b) Trogocytosis can only be observed by the transfer of H2Kd (red) onto EGFP+ (green) cells. The percent of double positive cells is based on the total of EGFP+ cells.

### Trogocytosis Occurs among Non-Blood Cells of the Solid Organs

Since our data has revealed that trogocytosis is more wide spread than previously reported, we sought to investigate if trogocytosis extends to non-blood tissues. The same chimeric animals analysed for blood-based trogocytosis in the previous section were further analysed for non-blood tissue based trogocytosis. Chimeric organs were divided and a portion was fixed for immunohistochemistry while the remaining tissue was treated with collagenase to generate single cells for flow cytometry analysis. Cells were stained for flow cytometry with anti mouse CD45antibody and anti H2Kd antibody. The CD45 antibody allowed us to remove any contaminating blood cells (CD45+) and CD45 negative cells were then analysed for EGFP and H2Kd. The same cytometry settings were used here as used for the blood cell analysis. Figure 6B and the accompanying Table 2 are representative from mouse #2 (50% coat colour chimerism and 80% blood cell chimerism). The total organ chimerism (% EGFP positive cells) is listed in Table 2 column 1 and the percentages of trogocytosis positive cells are listed in column 2. Trogocytosis positive cells from the non-blood organs (EGFP+/H2Kd+/CD45-) ranged from 1.0% of total EGFP+ cells in the spinal cord to 58% in the lung. However, we cannot determine from this experiment if the transferred H2Kd proteins that came from the BALB/c contribution was from the circulating blood cells or from the BALB/c cells within the organ. Closer examination of the results indicates that organs with high blood circulation such as the liver, lungs and heart have a higher percentage of EGFP cells co-expressing H2Kd then other tissues indicating a higher frequency of trogocytosis events. For example only 2% of the liver cells are EGFP+ (low chimerism) but 50% of these cells are also positive for H2Kd (high trogocytosis). In contrast, the spinal cord contained 33% EGFP+ cells (high chimerism) but only 1% co-expressed H2Kd and EGFP+ (low trogocytosis). These data suggests that blood cells are the source of the H2Kd antigen to the cells of organs.

**Figure 7:**
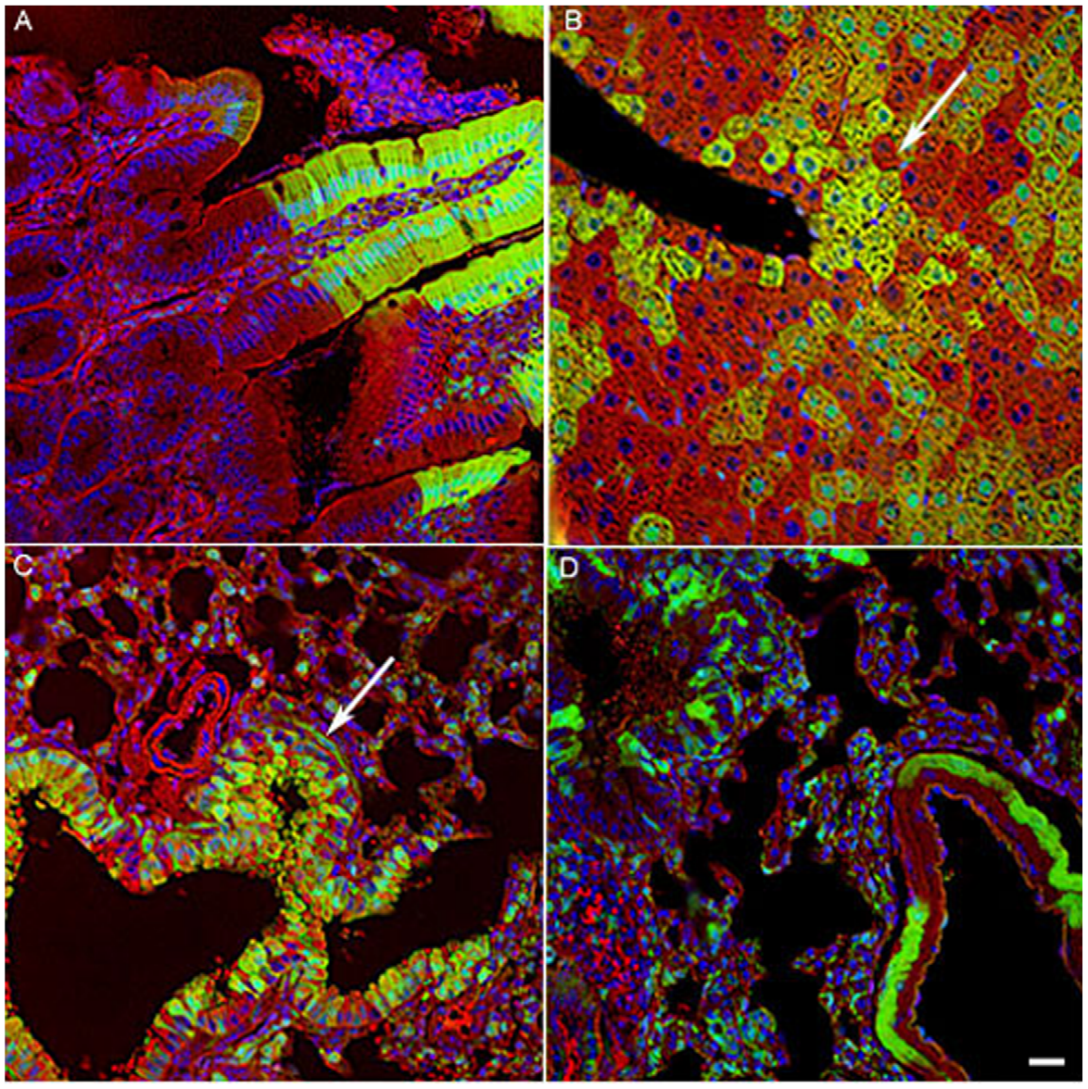
Trogocytosis in solid organs is observed in chimeric animals. A) Intestine, B) liver and (C and D) lung tissues, display distinct clonal boundaries between BALB/c and C57BL/6 cells. EGFP cells of C57BL/6 origin are green in colour while the H2Kd BALB/c cells are red. EGFP cells that have acquired H2Kd result in a yellow signal (white arrow). Scale bar = 50 µm

Analysis of the tissue sections from the chimeric mice confirms our flow cytometry results. Tissue sections were stained with an anti H2Kd antibody. Distinct boundaries between clonal contributions from the two different mouse strains are visible for intestine, liver and lung (Figure 7A-D). Distinct patches of yellow cells, resulting from the surface staining of transferred H2Kd with a red secondary antibody (Alexaflour 596) combined with green (EGFP+) cytoplasm, were observed in tissue sections indicative of the transfer of H2Kd to EGFP cells in the liver and the lung (Figure 7B,C arrow). Chimeric lung tissue with no trogocytosis is shown in Figure 7D.

## Discussion

Bone marrow transplantation using hematopoietic stem cells is a common procedure used to reconstitute the bone marrow and regenerate the immune system after full or partial myeloablative therapy to eradicate blood based cancers. Despite the widespread use of bone marrow transplants, there have not been any studies investigating the extent of cell fusion or trogocytosis between blood cells of the donor and host in the transplant setting. Because of the numerous reports of cell fusion and particularly fusion between blood cells and non-blood cells we investigated whether blood cells are capable of fusion with each other [13], [27]. Using a standard mouse model for hematopoietic stem cell engraftment we were able to demonstrate that >80% of the donor cells carried recipient surface MHC class I proteins suggesting a fusion event. Further investigation resulted in our concluding that cell-cell fusion was not the mechanism that could account for the high level of transfer nor could other mechanisms such as DNA uptake of apoptotic bodies by phagocytosising cells. The most parsimonious mechanism was trogocytosis.

The potential transfer of mismatched Class I HLA antigens between cells as identified in this study has implications for graft acceptance in the bone marrow transplant setting. Furthermore, in animal studies used to investigate the ability of blood cells to differentiate into non-blood tissues, the determination of successful stem cell differentiation or blood cell transdifferentiation relies heavily on the identification of cell surface markers on the regenerated tissue. Thus, the occurrence of hybrid cells due to cell fusion or surface antigen transfer between blood cells or blood cells and organs needs to be clarified when interpreting differentiation as well as transdifferentiation data. Furthermore, the recent report that the transfer mitochondria occurs between cells without cell fusion indicates that cell-cell interaction involves many novel mechanisms [28].

In this study we investigated the extent of trogocytosis between donor and recipient blood cells in the bone marrow transplant setting and between blood cells and non-blood cells in chimeric animals. Our study demonstrates that trogocytosis is more common than previously reported [26]. It has been demonstrated by others that dendritic cells, NK cells, T and B-cells are capable of acquiring MHC class1 antigens by trogocytosis at the immunological synapse. Here we have expanded on these studies to investigate the occurrence of surface protein transfer in the xenograft setting and its impact on our understanding of experimental disease models. We have clearly demonstrated that trogocytosis is not limited to antigen presenting cells as all donor hematopoietic blood cells, including the CD34+ stem and progenitor cells, acquired recipient MHC Class I molecules.

Mouse engraftment models have been the standard assay used to identify blood stem cells. Despite the long history of using murine models for intra-species and xenograft transplants, our observation that almost all of the donor blood cells acquired recipient MHC proteins in 100% of the engrafted mice, regardless of time post transplant (t = 7 days to t = 7 months), has not been previously reported. Our NOD/SCID mouse transplant protocol results in low numbers of human antigen presenting T and NK cells and B-cells and therefore, trogocytosis due to immune cell reactions cannot account for all of the events observed in this study [12], [29], [30], [31]. Although there was not a transfer of DNA during trogocytosis, our long-term studies and peripheral blood analysis demonstrate that trogocytosis is an ongoing event and was maintained in the recipient mice for 7 months. FISH analysis, karyotyping and the loss of cell surface markers after in vitro culturing confirmed that trogocytosis and not cell fusion was the cause of hybrid cells in a bone marrow transplant setting. Furthermore, using chimeric animals, we have demonstrated that both blood and non-blood tissues are involved in trogocytosis. Our observation that MHC-Class I antigens are involved implicates potential role of trogocytosis in graft acceptance.

In the bone marrow transplant setting, we were able to demonstrate that trogocytosis occurs in the bone marrow and not the peripheral blood, where the cells find themselves in close contact, a requirement for surface protein transfer. Therefore trogocytosis requires that the donor cells migrate to the bone marrow and engraft. In the clinical setting, trogocytosis would require the presence of recipient cells, a condition that is met in myeloablative conditioning of humans as donor cell infusion occurs before the patient becomes aplastic. Recipient bone morrow cells would also be present to participate in trogocytosis in reduced intensity and non-myeloablative conditioning regimes used for human bone marrow transplants.

Interestingly, our studies indicate that trogocytosis is unidirectional for MHC-Class I antigens, with proteins moving from the recipient cells to the donor cells. The widespread transfer of recipient MHC class1 molecules to donor blood cells and donor organs led us to question if other surface proteins are involved in trogocytosis as well. Recipient (mouse) CD45, but not recipient CD14 or CD41a, could be transferred to donor (human) cells. These observations, along with the chimera studies, demonstrate MHC Class I molecules are routinely transferred in the transplant setting, as are CD45 antigens, although the latter occurs to a lesser extent. Despite the fact that donor stem and progenitor cells (CD34+) are involved in trogocytosis, it is unlikely that these cells are the sole source of hybrid cells. Hematopoietic cells undergo extensive cell division before moving into the circulation and it is unlikely any recipient proteins acquired by trogocytosis at the stem cell level would be present on mature cells.

It has also been demonstrated by others that trogocytosis results in the transfer of proteins that can maintain their function on the receiving cells. For example, there is evidence that HLA-G can be transferred from a tumour cell to a healthy cell, conferring the same NK cell suppression function that it had on the tumour cell[32] and T-cells that acquired CD80 and HLA-DR from antigen presenting cells developed an antigen presenting-cell function [33]. The demonstration that HLA-G or CD80/HLA-DR transfer results in the establishment of function suggests that the transfer of MHC class I proteins from the recipient cells to the donor cells observed in our study may alter the way the donor cell is perceived by the host immune system [34]. We can speculate that the transfer of recipient MHC could confer a ‘recipient-self’ identity to the donor cells, thus protecting them from immune attack in the bone marrow transplant setting. Support for this hypothesis comes from the demonstration that the genetic regulation of NK cell-mediated rejection of bone marrow grafts is dependent on the MHC class I genes. It has been demonstrated that allogenic grafts are rejected because they fail to express the ‘self’ MHC recognised by the recipient NK cells. In one study, it was demonstrated that the introduction of a single transgene of the recipient MHC-class I gene into mismatched donor bone marrow cells resulted in the donor cells being able to present as ‘self’ MHC in the recipient, thereby altering the reaction of NK cells and preventing rejection [34].

Blood cells have therapeutic potential beyond the reconstitution of the bone marrow. Using different animal models of human disease, blood cells have been shown to give rise to neural cells, endothelial cells, striated muscle, hepatocytes and cardiac tissue[35], [36], [37]. It has been suggested that blood cells have the ability to transdifferentiate due to their ability to give rise to non-blood cells such as cardiomyocytes [38] or hepatocytes [3] while other studies have demonstrated that putative transdifferentiation events are actually a result of cell-cell fusion [23], [27]. Transdifferentiation-like events can also be linked to the possible presence of a rare pluripotent embryonic-like stem cell in the blood [39]. While it is possible that transdifferentiation events can be due to one or all of these mechanisms our study indicates that trogocytosis may account for some putative transdifferentiation events involving blood cells.

The chimera mouse model is ideal for the study of trogocytosis as there have been no reports of cell fusion in chimeras [40]. Furthermore, cell fusion that has been observed in the liver between hepatocytes and blood cells is initiated by tissue damage [41]. The lack of any induction of tissue damage in our chimera experiments allowed us to observe trogocytosis only. The data from the chimera studies suggests that blood flow within an organ and not the level of organ chimerism dictates the frequency of trogocytosis as organs with high blood flow had high levels of trogocytosis despite their levels of chimerism. For example liver had only 2% chimerism (2% EGFP+ cells) but 50% of these cells were involved in trogocytosis (EGFP+/H2Kd+) versus the spinal cord with 33% chimerism but only 1% of these cells were also H2Kd+.

It is evident that trogocytosis is becoming an important mechanism to consider for normal immune reactions, organ and bone marrow transplantation and experimental animal models for cell differentiation.

## Materials and Methods

### Ethics Statement

Collection of all human tissue samples were approved by the Research Ethics Boards of the University of Toronto and Mount Sinai Hospital, Toronto, Canada. Patients were required to read a patient information sheet and sign a consent form prior to any samples being collected. All samples were anonymous.

All animal studies were approved by the animal ethics board at Mount Sinai Hospital (AUP# 09-12-0021a-H).

### Blood Collection, Processing and Cryopreservation

Qualified hospital personnel, following protocols approved by the human ethics committee of the Mt. Sinai Hospital and the University of Toronto, collected the cord blood at the time of delivery. The BM or UCB samples were screened for HIV I/II, HTLV-I/II, Hepatitis B (HBs Ag), Hepatitis C (anti HVC), CMV and VDRL and written consent for collecting and processing umbilical cord blood was obtained at the time of registration.

The blood volume was reduced and the red blood cells removed with Pentaspan (starch) (DuPont, USA) as described previously [37]. All samples were cryopreserved prior to use by storage in liquid nitrogen.

### Isolation of an Enriched Stem Cell or Progenitor Cell Population

Human UCB or BM were used fresh or thawed and diluted drop wise in 10× volume, pelleted and resuspended in 0.1% BSA/HBSS. Lin- cells were isolated using a negative selection column according to the manufacturer's instructions (StemCell Technologies, Canada). The antibody mix contained a set of lineage specific surface markers found on mature hematopoietic cells; CD2, CD3, CD14, CD16, CD19, CD24, CD56, CD66 and glycophorin A. Following isolation, the Lin- cells or the Lin+ cells were used directly in the SRC-assay.

### Long Term Cell-Initiating Cell Assay (LTC-IC) Repopulating Assay and Colony Forming Unit Assay (CFU)

A pre-made methylcellulose based colony assay medium (Stem Cell Technologies, Canada) was used as described by the manufacturer. The medium is formatted to grow mouse (M3434) or human (H4344) primitive progenitors. Total nucleated cells from bone marrow aspirates were stained with antibodies to H2Kd and HLA-ABC and sorted by flow cytometry. Sorted cells were plated at 5000 and 2000 cells per 3 ml of medium. All populations were plated in duplicate and scored at days 12, 14, 16 and 18. Day 16 data is represented in Table 1. Cells were also tested for primitive progenitor cells by LTC-IC protocol as described by the manufacturer (Stem Cell Technologies, Canada). Briefly, cells were plated on M2-10B4 fibroblasts (Stem Cell Technologies, Canada) and cultured for 5 weeks with half medium changes weekly. Cells were then collected and plated in a CFU assay (H4344) and scored at day 18 for colonies.

### NOD.CB17-Prkdc^SCID^ Mouse Engraftment

Non-obese severe combined immune deficient NOD.CB17-Prkdc^SCID^ mice (Jackson Labs, Bar Harbour, Maine, USA) were used to test the engraftment potential of human blood cells [24]. All experiments followed established protocols and received animal ethics approval at Mt. Sinai Hospital. The mice were irradiated 2 hours before engraftment at 360 Rads using a Cs^137^ source. The mice were then injected via the tail vein with 100 µl of cell solution. Survival rate was >80% per experiment. At different time intervals, animals were euthanized and the bone marrow was flushed from the femurs. Cells were washed in PBS and the cell pellets were subjected to Red Blood Cell Lysis Buffer for 3 minutes and washed again.

### Flow Cytometry

#### A: Bone marrow analysis

Mice were sacrificed from 7 days to 7 months post transplantation, the femurs excised and flushed using 1 ml of PBS containing 2% FBS (2% FBS/PBS). Cells were treated with RBC Lysis Buffer and washed once with 2% FBS/PBS and pelleted. The cell pellet was resuspended at 1 million cells/ml and immunostained. After the antibody was added, cells were incubated on ice for 30 min. washed twice in 2% FBS/PBS and resuspended at 1 million cells per 0.2 ml and immediately analysed by flow cytometry with a Coulter-Epics Flow cytometer (Coulter, Burlington, Canada). Isotype controls were used in all cases. Peripheral blood was collected for analysis by heart puncture before bone marrow was collection.

#### B: Chimera analysis

To generate single cell suspensions from mouse organs, mice were perfused with 5% FBS/HBSS and the organs dissected and minced on ice. These minced tissues were washed with PBS and incubated at 37°C in an enzyme solution containing 1.5 mg/ml collagenase + 0.25% trypsin in PBS for 10–30 minutes depending on the size of the tissue fragment. After incubation, DMEM containing 5% FBS was added to the tissues that were then triturated with a large bore pipette so not to shred the cells. The large fragments were allowed to settle (30 sec) and the single cells in suspension were collected. This single cell suspension was passed through a 70 micron mesh filter and at this point was ready for FACS immunostaining as outlined above. The range of EGFP fluorescence intensity in chimeric mice was variable, so in order to clearly display the full range of EGFP expressing cells required that the detectors for EGFP were set to capture and display all EGFP positive cells at the expense of the negative cells being pushed up against the Y-axis. Original histograms displaying percentages of cells for each quadrant are shown in the figures. The settings for the detectors are shown in Table 3.

**Table 3:** Flow cytometry detector settings.

SENSORS	FS	PMT1	PMT2	PMT3	PMT4	PMT5
Volts	250	295	1400/**1050**	1050	1090	1350
I Gain	7.5	10	10	10	10	7.5
P Gain	2		3	10	10	
Fluorescence Compensation						
X	PMT1	PMT2	PMT3	PMT4	PMT5	
Y						
PMT1						
PMT2			1.5/**0.2**	0		
PMT3		8.9/**56.9**				
PMT4						
PMT5			8			

The Coulter-Epics Flow cytometer (Coulter, Burlington, Canada) settings for EGFP detection in chimeric tissues are displayed. Bold numbers are for EGFP and PE detection. All others are for FITC and PE detection.

### Antibodies

Primary antibodies used: H2Kd (BD-Pharmingen, USA), mouse CD45 (BD-Pharmingen, USA), human CD45 (J33) (Immunotech, France), HLA-ABC (Immunotech, France), human CD34 (Immunotech, France), mouse CD14 (BD-Pharmingen, USA), human CD14 (Immunotech, France), mouse CD41a (BD-Pharmingen, USA), human CD41a (BD-Pharmingen, USA). Secondary antibodies conjugated with Alexafluor 596 (Red) or Alexafluor 488 (Green) were used.

### Fluorescent In-Situ Hybridization

Pan centromeric chromosome paint for human (cat# 1695) and mouse (cat# 1697) from StarFISH (Cambio, England) was used for fluorescent in-situ hybridization (FISH). Cells were fixed in 3∶1 ethanol:acetic acid, dehydrated in an ethanol series, mounted on slides and air dried FISH was performed according to the manufacturer's protocol (StarFISH, Cambio, England).

### Chimera Production

Fluorescent variants of C2-C57BL/6NTac ES cells were generated by the random integration of EGFP transgene driven by a CMV immediate early enhancer coupled to the chicken β-actin promoter and first intron (pCAG) using co-electroporation with a circular selectable marker pPGK Puro using standard ES cell culture protocols[42]. Chimeric mice were produced by aggregation of C57BL/6 EGFP ES cell line #12 with BALB/cAnNCrl morula stage embryos as described[42], [43]. Chimeras were identified by a mixed black and albino coat colour. Chimeras were sacrificed at 8-weeks of age and tissues were processed for immunohistochemistry or flow cytometry.

### Immunohistochemistry

Tissues were fixed in 10% buffered formalin (Fisher Scientific, USA) for 120 min, at 4°C, washed in PBS (Phosphate Buffered Saline), stored in 70% ethanol, dehydrated in graded ethanol series, cleared in toluene, immersed in paraffin at 65°C, embedded into paraffin blocks and sectioned into 5 µm sections. Sections were deparaffinized 2×5 min in xylene. Nonspecific binding was blocked with 10% serum in PBS containing 0.1% Triton X-100 (Sigma, USA) for 240 min at room temperature, followed by a brief wash in PBS. Primary antibody was applied (solution 1∶50–1∶100) for overnight incubation at 4°C. Untreated mouse tissue sections and sections with omitted primary or secondary antibody were used as negative controls. The slides were washed 5×15min in PBS, the secondary antibody was applied at 1∶200 dilution for 60 min at room temperature, and then washed again 6×15 min in PBS. Finally, the slides were mounted in 50% glycerol in PBS with DABCO (Sigma, USA) at 100 mg/ml.

The slides were examined on a Zeiss Axioplan Photomicroscope equipped with epifluorescent ultraviolet light and corresponding excitation and barrier filters. Pictures were taken on a Delta Vision wide-field, optical sectioning microscope workstation capable of recording three-dimensional images of fluorescently labelled specimens (Issaquah, Washington). The station includes∶ an Olympus IX-70 inverted fluorescence microscope with custom optical filters, and precision XYZ motorized stage, O2 Silicon Graphics computer work station with image collection and deconvolution software.

## Supporting Information

Figure S1 Confirmation of the specificity of the antibodies used for FACS analysis.
(0.96 MB TIF)

## References

[1] McCulloch EA, Till JE (1960). The radiation sensitivity of normal mouse bone marrow cells, determined by quantitative marrow transplantation into irradiated mice.. Radiat Res.

[2] Till JE, Mc CE (1963). Early repair processes in marrow cells irradiated and proliferating in vivo.. Radiat Res.

[3] Grompe M (2005). Bone marrow-derived hepatocytes.. Novartis Found Symp.

[4] Rampon C, Weiss N, Deboux C, Chaverot N, Miller F (2008). Molecular mechanism of systemic delivery of neural precursor cells to the brain: assembly of brain endothelial apical cups and control of transmigration by CD44.. Stem Cells.

[5] Kroon E, Martinson LA, Kadoya K, Bang AG, Kelly OG (2008). Pancreatic endoderm derived from human embryonic stem cells generates glucose-responsive insulin-secreting cells in vivo.. Nat Biotechnol.

[6] Yamada S, Nelson TJ, Crespo-Diaz RJ, Perez-Terzic C, Liu XK (2008). Embryonic stem cell therapy of heart failure in genetic cardiomyopathy.. Stem Cells.

[7] Maitra B, Szekely E, Gjini K, Laughlin MJ, Dennis J (2004). Human mesenchymal stem cells support unrelated donor hematopoietic stem cells and suppress T-cell activation.. Bone Marrow Transplant.

[8] Tisato V, Naresh K, Girdlestone J, Navarrete C, Dazzi F (2007). Mesenchymal stem cells of cord blood origin are effective at preventing but not treating graft-versus-host disease.. Leukemia.

[9] Gorin NC, Piantadosi S, Stull M, Bonte H, Wingard JR (2002). Increased risk of lethal graft-versus-host disease-like syndrome after transplantation into NOD/SCID mice of human mobilized peripheral blood stem cells, as compared to bone marrow or cord blood.. J Hematother Stem Cell Res.

[10] Shlomchik WD (2007). Graft-versus-host disease.. Nat Rev Immunol.

[11] Rogers IM, Yamanaka N, Casper RF (2008). A simplified procedure for hematopoietic stem cell amplification using a serum-free, feeder cell-free culture system.. Biol Blood Marrow Transplant.

[12] Madlambayan GJ, Rogers I, Purpura KA, Ito C, Yu M (2006). Clinically relevant expansion of hematopoietic stem cells with conserved function in a single-use, closed-system bioprocess.. Biol Blood Marrow Transplant.

[13] Oren-Suissa M, Podbilewicz B (2007). Cell fusion during development.. Trends Cell Biol.

[14] Zhou Q, Melton DA (2008). Extreme makeover: converting one cell into another.. Cell Stem Cell.

[15] Rechavi O, Goldstein I, Kloog Y (2009). Intercellular exchange of proteins: the immune cell habit of sharing.. FEBS Lett.

[16] Murry CE, Soonpaa MH, Reinecke H, Nakajima H, Nakajima HO (2004). Haematopoietic stem cells do not transdifferentiate into cardiac myocytes in myocardial infarcts.. Nature.

[17] Sussman MA, Murry CE (2008). Bones of contention: marrow-derived cells in myocardial regeneration.. J Mol Cell Cardiol.

[18] Shi D, Reinecke H, Murry CE, Torok-Storb B (2004). Myogenic fusion of human bone marrow stromal cells, but not hematopoietic cells.. Blood.

[19] Hudrisier D, Riond J, Garidou L, Duthoit C, Joly E (2005). T cell activation correlates with an increased proportion of antigen among the materials acquired from target cells.. Eur J Immunol.

[20] Sharrow SO, Mathieson BJ, Singer A (1981). Cell surface appearance of unexpected host MHC determinants on thymocytes from radiation bone marrow chimeras.. J Immunol.

[21] Tabiasco J, Espinosa E, Hudrisier D, Joly E, Fournie JJ (2002). Active trans-synaptic capture of membrane fragments by natural killer cells.. Eur J Immunol.

[22] Riond J, Elhmouzi J, Hudrisier D, Gairin JE (2007). Capture of membrane components via trogocytosis occurs in vivo during both dendritic cells and target cells encounter by CD8(+) T cells.. Scand J Immunol.

[23] Alvarez-Dolado M, Pardal R, Garcia-Verdugo JM, Fike JR, Lee HO (2003). Fusion of bone-marrow-derived cells with Purkinje neurons, cardiomyocytes and hepatocytes.. Nature.

[24] Madlambayan GJ, Rogers I, Kirouac DC, Yamanaka N, Mazurier F (2005). Dynamic changes in cellular and microenvironmental composition can be controlled to elicit in vitro human hematopoietic stem cell expansion.. Exp Hematol.

[25] Vormoor J, Lapidot T, Pflumio F, Risdon G, Patterson B (1994). Immature human cord blood progenitors engraft and proliferate to high levels in severe combined immunodeficient mice.. Blood.

[26] Davis DM (2007). Intercellular transfer of cell-surface proteins is common and can affect many stages of an immune response.. Nat Rev Immunol.

[27] Johansson CB, Youssef S, Koleckar K, Holbrook C, Doyonnas R (2008). Extensive fusion of haematopoietic cells with Purkinje neurons in response to chronic inflammation.. Nat Cell Biol.

[28] Spees JL, Olson SD, Whitney MJ, Prockop DJ (2006). Mitochondrial transfer between cells can rescue aerobic respiration.. Proc Natl Acad Sci U S A.

[29] Kalberer CP, Siegler U, Wodnar-Filipowicz A (2003). Human NK cell development in NOD/SCID mice receiving grafts of cord blood CD34+ cells.. Blood.

[30] Wege AK, Melkus MW, Denton PW, Estes JD, Garcia JV (2008). Functional and phenotypic characterization of the humanized BLT mouse model.. Curr Top Microbiol Immunol.

[31] Morrison SJ, Uchida N, Weissman IL (1995). The biology of hematopoietic stem cells.. Annu Rev Cell Dev Biol.

[32] Caumartin J, Favier B, Daouya M, Guillard C, Moreau P (2007). Trogocytosis-based generation of suppressive NK cells.. Embo J.

[33] Sabzevari H, Kantor J, Jaigirdar A, Tagaya Y, Naramura M (2001). Acquisition of CD80 (B7-1) by T cells.. J Immunol.

[34] Ohlen C, Kling G, Hoglund P, Hansson M, Scangos G (1989). Prevention of allogeneic bone marrow graft rejection by H-2 transgene in donor mice.. Science.

[35] Kogler G, Sensken S, Airey JA, Trapp T, Muschen M (2004). A new human somatic stem cell from placental cord blood with intrinsic pluripotent differentiation potential.. J Exp Med.

[36] Krause DS, Theise ND, Collector MI, Henegariu O, Hwang S (2001). Multi-organ, multi-lineage engraftment by a single bone marrow-derived stem cell.. Cell.

[37] Rogers I, Yamanaka N, Bielecki R, Wong CJ, Chua S (2007). Identification and analysis of in vitro cultured CD45-positive cells capable of multi-lineage differentiation.. Exp Cell Res.

[38] Deb A, Wang S, Skelding KA, Miller D, Simper D (2003). Bone marrow-derived cardiomyocytes are present in adult human heart: A study of gender-mismatched bone marrow transplantation patients.. Circulation.

[39] Ratajczak MZ, Machalinski B, Wojakowski W, Ratajczak J, Kucia M (2007). A hypothesis for an embryonic origin of pluripotent Oct-4(+) stem cells in adult bone marrow and other tissues.. Leukemia.

[40] Kidder BL, Oseth L, Miller S, Hirsch B, Verfaillie C (2008). Embryonic stem cells contribute to mouse chimeras in the absence of detectable cell fusion.. Cloning Stem Cells.

[41] Zhou P, Hohm S, Olusanya Y, Hess DA, Nolta J (2009). Human progenitor cells with high aldehyde dehydrogenase activity efficiently engraft into damaged liver in a novel model.. Hepatology.

[42] Nagy A, Gertsenstein M, Vintersten K, Behringer R (2003). Manipulating the Mouse Embryo..

[43] Tanaka M, Hadjantonakis AK, Nagy A (2001). Aggregation chimeras. Combining ES cells, diploid and tetraploid embryos.. Methods Mol Biol.

