# Supplementary figures and images for: Bone Marrow Transplantation Results in Human Donor Blood Cells Acquiring and Displaying Mouse Recipient Class I MHC and CD45 Antigens on Their Surface
Source: PLoS One. 2009 Dec 31;4(12):e8489. doi: 10.1371/journal.pone.0008489 (PMC2796175; doi:10.1371/journal.pone.0008489)

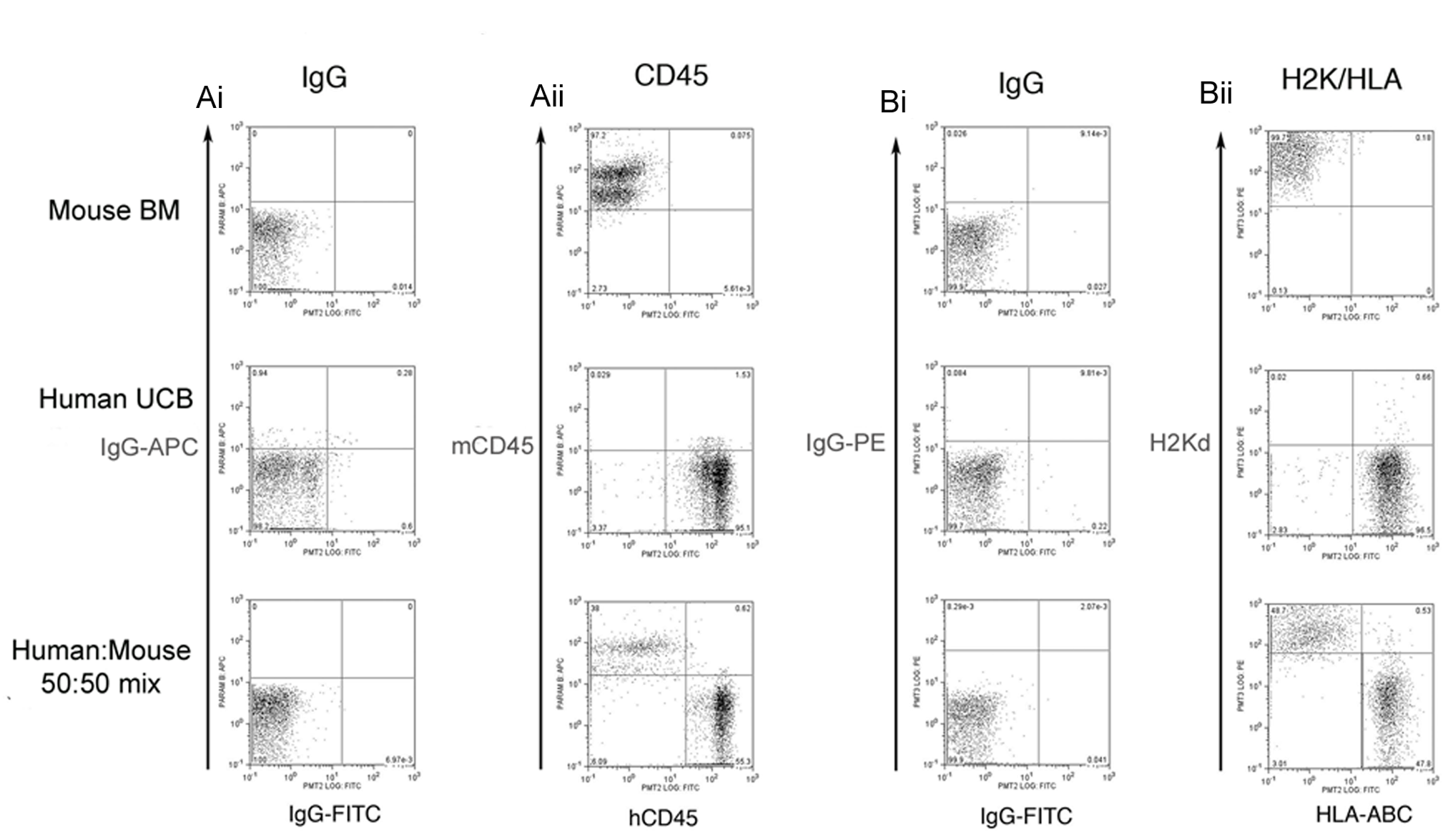

Supplement: Figure S1 — Confirmation of the specificity of the antibodies used for FACS analysis. (0.96 MB TIF) [file pone.0008489.s001.tif]
